# Health care burden and mortality of acute on chronic liver failure in Thailand: a nationwide population-based cohort study

**DOI:** 10.1186/s12913-022-07574-6

**Published:** 2022-02-07

**Authors:** Sakkarin Chirapongsathorn, Kittiyod Poovorawan, Ngamphol Soonthornworasiri, Wirichada Pan-ngum, Amnart Chaiprasert, Kamthorn Phaosawasdi, Sombat Treeprasertsuk

**Affiliations:** 1 Division of Gastroenterology and Hepatology, Department of Medicine, Phramongkutklao Hospital and College of Medicine, Bangkok, Thailand; 2 Department of Clinical Tropical Medicine, Faculty of Tropical Medicine, Mahidol University, Bangkok, Thailand; 3 Department of Tropical Hygiene, Faculty of Tropical Medicine, Mahidol University, Bangkok, Thailand; 4 Division of Nephrology, Department of Medicine, Phramongkutklao Hospital and College of Medicine, Bangkok, Thailand; 5 Division of Gastroenterology, Vichaiyut Hospital, Bangkok, Thailand; 6 Division of Gastroenterology, Department of Medicine, Chulalongkorn University, Bangkok, Thailand

**Keywords:** Cirrhosis, Acute on chronic liver failure (ACLF), Costs analysis, Liver failure, Public health, Portal hypertension, Nationwide study, Burden, Health care delivery, Hospitalization

## Abstract

**Background:**

Accurate population-based data are required concerning the rate, economic impact, and long-term outcome from acute on chronic liver failures (ACLF) in hospitalized patients with cirrhosis.

We aimed to discover time trends for the epidemiology, economic burden, and mortality of ACLF in Thailand.

**Methods:**

We conducted a nationwide, population-based, cohort study which involved all hospitalized patients with cirrhosis in Thailand during the period between 2009 and 2013, with data from the National Health Security Office. ACLF was defined by two or more extrahepatic organ failures in patients with cirrhosis. Primary outcomes were trends in hospitalizations, hospital costs, together with inpatient mortality.

**Results:**

The number of ACLF hospitalizations in Thailand doubled between 3185 in 2009 and 7666 in 2013. The average cost of each ACLF hospitalization was 3.5-fold higher than for cirrhosis ($ 1893 versus $ 519). The hospital is paid using a diagnosis-related group (DRG) payment system that is only 15% of the average treatment costs ($ 286 from $ 1893). The in-hospital fatality rate was 51% for ACLF while the additional fatality rate was 85% up to 1 year. The ACLF organ failure trends indicated sepsis with septic shock and renal failure as the majority proportion. Age, the number and types of organ failure and male sex were predictors of ACLF death.

**Conclusions and relevance:**

Cirrhosis and ACLF both represent substantial and increasing health and economic burdens for Thailand. These data can assist national health care policy stakeholders to target high-risk patients with cirrhosis for care.

**Supplementary Information:**

The online version contains supplementary material available at 10.1186/s12913-022-07574-6.

Acute and chronic liver diseases not only affect costs but are a burden to the worldwide health care system [1]. All chronic liver diseases show progression to cirrhosis over time, and this is a late stage of liver fibrosis which causes various complications that significantly increase mortality rates. Global cirrhosis deaths are estimated to be greater than 1.3 million per year [2]. Cirrhosis also impacts health care costs due to the need of highly specialized care with significant resource utilization for management of complications including intensive care [3]. Hospitalized patients cause an increasing economic burden with more admissions, and readmissions resulting in longer hospital stays especially for decompensated cirrhosis [3, 4].

Although there have been evidence-based quality improvements in cirrhotic care, in decompensated cirrhosis hospital patients mortality rate has remained high [5]. Among hospitalized patients with cirrhosis, those who progress to acute on chronic liver failure (ACLF) experience rapid multi-organ deteriorations resulting in high risk of mortality while consuming high intensive care resources. ACLF is a frequent complication in hospitalized patients with cirrhosis, leading to high short-term mortality risk [6, 7]. Recent studies have reported high short-term (28-day to 30-day) mortality in patients with ACLF. Although, short-term mortality rate can vary from 22 to 73%, this depends on the number of failed organs, geographic location, and definition used by Western experts (US and Europe) and Asian countries for diagnosing ACLF [7–9]. Epidemiological ACLF data are heterogeneous which are difficult to compare. Efforts have tried to understand ACLF’s natural history and homogenies in order to compare the current evidence, which includes prevalence, pathogenesis of disease, precipitating factors, and outcomes. Furthermore, ACLF’s health care burden and economic impact have not been thoroughly assessed. Knowledge of disease burden using correct population-based data are required for public health policy planning assessment and resource assignment.

Our primary objective for this study, was estimation of the number of hospitalizations, together with associated costs and economic impacts, and patients with ACLF’ mortality between 2009 and 2013 in Thailand, using the database of the National Health Security Office. The secondary objectives were to investigate patient and liver disease characteristics which dominate markings of ACLF health care use and identification of the number and types of organ failure causing inpatient mortality.

## Materials and methods

### Data collection

All patient required data were resourced from the National Health Security Office. Data were extracted from national inpatient databases between 2009 and 2013 which included all stakeholders under the Thai Ministry of Public Health that incorporated representative administrative data sets with total population of 49.1 million. The databases included those resourced from nationwide hospitalizations. The National Health Security Office database comprised 28,294,685 individual patient discharge records between 2009 and 2013. Each record included a primary as well as other secondary discharge diagnoses, demographic data, procedure codes, mortality status, hospitalized inpatient mortalities, and various other data concerning the hospitals such as location. Each data set observation was an individual hospitalization episode which included admission of a patient and ended with discharge or mortality.

### Cohort selection

Hospitalized patients with cirrhosis were identified using one or more of these: International Classification of Diseases, 10th Revision, Clinical Modification (ICD-10-CM) codes: spontaneous bacterial peritonitis K65.2, alcoholic cirrhosis K70.30, esophageal varices without bleeding I85.00, esophageal varices with bleeding I85.01, varices in diseases classified elsewhere with/without bleeding I85.10, I85.11, hepatorenal syndrome K76.7, cirrhosis of liver without mention of alcohol K74.0, and hepatic encephalopathy K72.90, K72.91. We tested validity of the ICD-10, World Health Organization (WHO) Version for 2016 codes with an administrative database from Phramongkutklao Hospital by using a medical-linked system on the hospital database (using 100 randomly selected medical records) to identify patients. This set of ICD-10 codes, from the WHO Version for 2016 identified patients with cirrhosis with high accuracy (sensitivity 92% and specificity 94%). Ascites (R18.8) did not change the accuracy of cirrhosis diagnosis [4, 10]. Other exposure variables included age, hospital size, sex, and geographic location within the country. Mortality rate was retrieved from the death certificate database. ACLF was defined by at least two extrahepatic organ failures (cardiovascular, renal, respiratory, or cerebral) in patients with cirrhosis. Since our database had no laboratory data, this definition was selected to mirror ACLF grade 1 following the CANONIC (CLIF Acute on Chronic Liver Failure in Cirrhosis) study criteria and to connote the significantly higher mortality rate as shown by the North American Consortium for Study of End-stage Liver Disease [7]. ACLF hospitalizations were identified by using any one of these ICD-10-CM diagnoses which were from two different organ system groups: pulmonary artery/wedge pressure, arterial line, cardiovascular failure defined by using codes for central venous pressure, septic shock, severe sepsis; renal failure defined by using codes for hemodialysis, acute kidney failure; respiratory failure defined by using codes for mechanical ventilation; and cerebral failure defined by using codes for hepatic coma [11]. The diagnostic codes related to these diagnoses are summarized in Supporting Table S1 [4, 10, 11].

### Ethical consideration

This study was done with approval from The Gastroenterological Association of Thailand in collaboration with The National Health Security Office, Thailand. The research protocol was approved by the Institutional Review Board, Faculty of Tropical Medicine, Mahidol University (MUTM 2014-056-01).

### Statistical analysis

Data were analyzed using SPSS Statistics (IBM, Armonk, NY). Once all the cirrhosis and ACLF hospitalizations were identified, the suitable statistical procedures were used to compute nationwide estimates of inpatient health care costs, the number of hospitalizations, inpatient fatalities and survival status. Data were summarized as median (interquartile range) or mean (range) for continuous outcomes or as counts and percentages for categorical outcomes. Normal distributions were checked by visual inspection. Wilcoxon rank sum tests were used for continuous data and chi-square or Fisher exact tests were used for categorical data to ascertain differences between groups. Two-tailed *P* values were reported, with P less than 0.05 considered as statistically significant. The C statistic was computed for the discriminatory model performance using significant univariate and important clinical variables, which were then incorporated into the multivariate model. Cost was estimated with inflation-adjusted to 2020 Thai Baht currency based on cost-to-charge ratios from the Diagnosis-related Group (DRG), an essential payment method in universal healthcare coverage, of the Universal Coverage Scheme’s reimbursement system. DRG is the inpatient prospective payment system which categorizes hospitalization costs to determine how much to pay. Secondary outcomes included number and types of organ failure as in-hospital death predictors for an ACLF hospitalization.

## Results

### Characteristics of cirrhosis and ACLF hospitalization

The number of cirrhosis hospitalizations had increased every year from 2009 (*n* = 45,015) to 2013 (*n* = 58,113). Nearly a half of those hospitalizations were for alcohol-associated cirrhosis (48.6%) and the proportion for spontaneous bacterial peritonitis involvement was 29.2% of total hospitalizations. Among every discharge, the proportion who met the ACLF criteria increased almost double between 7.1% (*n* = 3185) in 2009 and 13.2% (*n* = 7666) in 2013. During the timeline of ACLF, there were no significant changes concerning patient characteristics comprising age, sex, hospital level as well as geographic distribution on hospitalized cirrhosis and patients with ACLF.

Table 1 shows the patient demographic and disease characteristics of discharges which met the study inclusion criteria between 2009 and 2013.

**Table 1:** Demographic and disease characteristics of hospitalized patients with cirrhosis and ACLF between 2009 and 2013

Characteristic	
All cirrhosis patients (including ACLF)	186,051
Age (mean ± sd)	52.1 ± 15.8
Male (%)	67
Cirrhosis etiology (%)	
Viral	7
Alcohol	44
Nonalcohol/nonviral	49
Cirrhosis complications (%)	
Hepatic encephalopathy	22
Esophageal varices	9
Hepatorenal syndrome	1
Spontaneous bacterial peritonitis	33
Sepsis (%)	8
Length of hospital stay (days) (mean ± sd)	6.4 ± 10.2
**ACLF subgroup**	22,950
Age (mean ± sd)	54.4 ± 14.2
Cirrhosis etiology (%)	
Viral	19
Alcohol	41
Nonalcohol/nonviral	40
Rate of ACLF in cirrhosis (%)	12.3 (22,950/186051)
Rate of ACLF in organ failure (%)	
Respiratory	70
Cardiovascular	64
Renal	62
Cerebral	18
Sepsis (%)	27.9
Length of hospital stay (days) (mean ± sd)	8.7 ± 13.4

We identified 22,950 patients with ACLF who satisfied study inclusion criteria. Overall, most patients with ACLF were admitted in Northeastern and Central regions of Thailand, 10.8% were community hospital admissions (less than 120 beds), 37.8% were intermediate-level hospital admissions (121-500 beds) and 51.4% were referral hospital admissions (more than 500 beds). The mean age was 54 years, 67% were male, and 100% were Asian (Thai descent). The most common cirrhosis complication leading to hospitalization documented was spontaneous bacterial peritonitis, and this was followed by hepatic encephalopathy. The period of hospital stay remained fairly constant from 2009 to 2013, which was higher for ACLF (9 days) in comparison with cirrhosis without ACLF (4 days). Table 2 illustrates the characteristics of ACLF patients hospitalized compared with those with cirrhosis.

**Table 2:** Characteristics of patients hospitalized with ACLF versus cirrhosis without ACLF

Characteristic	Non-ACLF (Cirrhosis) (***n*** = 163,101)	ACLF (***n*** = 22,950)
Age (mean ± sd)	51.9 ± 15.9	53.7 ± 14.8
Male (%)	67	67
Hospital level (%)		
Community hospital and Intermediate-level hospital	71	48
Referral hospital	29	52
Cirrhosis etiology (%)		
Viral	5	19
Alcohol	44	41
Nonalcohol/nonviral	51	40

There were no differences found in age and sex between ACLF and cirrhosis.

Sepsis and septic shock were substantial trends within factors related to ACLF, which remarkably increased the proportion of septic shock from 2012 to 2013 (28.2% in 2009, 27% in 2010, 26.2% in 2011 to 39.2% in 2012 and 58.3% in 2013).

The proportion and type of organ failure in ACLF remained notably constant throughout the study period, while respiratory failure averaged 70% and renal failure averaged 58.4%. Among renal failure patients, approximately 7.2% received hemodialysis and 3% received peritoneal dialysis. Overall, 77.4% had two organ failures, 19.3% had three organ failures, and 3.1% had four organ failures.

### Outcomes of cirrhosis and ACLF hospitalization

The in-hospital mortality rate was 51.7%. The 30-day mortality rate was nearly equal between 67.9% in 2009 and 68.3% in 2013 for ACLF and between 32.1% in 2009 and 31.7% in 2013 for cirrhosis. The 90-day mortality rate also was almost equal between 74.1% in 2009 and 75.1% in 2013 for ACLF and between 25.9% in 2009 and 24.9% in 2013. Throughout the study period, we found the mortality rate was 85.6% for ACLF. Sepsis and bacterial infection were the most common causes of mortality, followed by liver-related death and hepatocellular carcinoma in ACLF. Readmission rate among ACLF was only 3.4% and 30-day readmission rate was just 1%.

Table 3 represents predictors of 30-day mortality in ACLF, including age (hazard ratio [HR], 1.014; 95% confidence interval [CI]: 1.013-1.015), and male sex (HR, 1.075; 95% CI 1.056-1.095).

**Table 3:** Determinants of 30-day mortality in ACLF by multivariate analysis

	Adjusted HR (95% CI)	***P***
Age	1.014 (1.013-1.015)	< 0.0001
Male	1.075 (1.056-1.095)	< 0.0001
Hospital level		
Community hospital	Reference	
Intermediate-level hospital	1.325(1.297-1.354)	< 0.0001
Referral hospital	1.255(1.227-1.283)	< 0.0001
**Number and type of organ failure**		
Cirrhosis or one organ failure	Reference	
Two organ failures		
Respiratory + cardiovascular	4.686(4.534-4.843)	< 0.0001
Respiratory + renal	5.436(5.271-5.605)	< 0.0001
Respiratory + cerebral	4.187(3.867-4.535)	< 0.0001
Cardiovascular + renal	2.184(2.081-2.293)	< 0.0001
Cardiovascular + cerebral	1.141(1.030-1.264)	0.012
Renal + cerebral	2.333(2.129-2.556)	< 0.0001
Three organ failures	5.416(5.215-5.624)	< 0.0001
Four organ failures	6.277(5.331-7.391)	< 0.0001

Compared with patients, who were admitted to a community hospital, risk of death in patients admitted to an intermediate-level hospital (HR, 1.325; 95% CI 1.297-1.354) and a referral hospital (HR, 1.255; 95% CI 1.227-1.283) were significantly higher. As anticipated, the risk of death in patients with three organ failures (HR, 5.416; 95% CI 5.215-5.624) and four organ failures (HR, 6.277; 95% CI 5.331-7.391) were significantly higher compared with patients with no, one, or two organ failures. Among patients with two organ failures, respiratory together with renal failure showed the highest risk of death (HR, 5.436; 95% CI 5.271-5.605).

### Costs of cirrhosis and ACLF hospitalization

From 2009 to 2013, the yearly hospitalization costs for ACLF and cirrhosis increased from $ 29 million to $ 37 million. The mean cost per hospitalization for cirrhosis minimally increased from $ 659 to $ 661, while the mean hospitalization cost for ACLF decreased from $ 1947 to $ 1892. The mean cost per hospitalization for ACLF was 3.5-fold more than that for cirrhosis ($ $1893 versus $ 519). The hospital gets payment from the Universal Coverage Scheme by using a diagnosis-related group (DRG) payment system which is only 15% of the average treatment costs ($ 286 from $ 1893). Details of costs of ACLF and cirrhosis are summarized in Supporting Table S2.

## Discussion

This nationwide study showed the ACLF burden in the health care delivery system. Patients with cirrhosis who developed ACLF were at risk of death which represented a relatively high health care cost. Our nationwide population-based study revealed five key findings. First, the number of hospitalizations for cirrhosis rose and the proportion of ACLF rose by nearly double. Second, alcohol-related cirrhosis was the major identified risk in patients with cirrhosis and ACLF. Third, the mean ACLF proportion in cirrhosis was about 12.3% which was mainly associated with cardiovascular, respiratory, and renal failure. Fourth, higher number and type of organ failures are stronger risk of death in patients with ACLF. Risk of death in patients with two or more organ failures was significantly more than those with no or one organ failure and cardiovascular, respiratory, and kidney failures were strong risks to predict mortality in ACLF. Five, the economic burden of ACLF is credibly more when it develops because hospitals were reimbursed from government less than expectation.

The major obstacle for studying epidemiology of ACLF has several definitions. Currently there are three ACLF definitions; the first is from the CANONIC study in Europe [7]. It reported that ACLF contributed 30% of hospitalizations with 1343 cirrhotic patients and 28-day mortality varied from 22 to 73%. The North American Consortium for Study of End-Stage Liver Disease (NACSELD), a consortium comprising tertiary-care hepatology centers in North America, study defined ACLF as two organs’ failure in patients with cirrhosis [8]. In that study 24% of patients developed ACLF and 23% of patients died during their hospitalization and 30-days of follow up. However, another United States data from the National Inpatient Sample (NIS) showed lower rate of ACLF (1.5-5% of cirrhosis hospitalizations) [11]. The Asia-Pacific region definition was reported in a consensus paper of the Asian Pacific Association for the Study of Liver (APASL) 2014 meeting [12]. That study used retrospective and prospective enrollment of 1363 patients. The 1-month mortality rate was high at 51% compared with the worldwide rate. The divergence was associated with differences in studies’ populations (tertiary centers versus nationwide, community versus academic hospital), patient characteristics (acute decompensation of cirrhosis in CANONIC, infection-related hospitalization of patients with cirrhosis in NACSELD, versus all patients with cirrhosis in NIS and APASL), and ACLF definition which included use of laboratory date in the CANONIC study. Concerning this discrepancy, we determined to conduct another nationwide population based data using the same ALCF definition reference from the NIS study [11]. To the best of our knowledge, this is the first report about nationwide ACLF data outside of North America. The ACLF prevalence in the Thailand health care database was more and trended to increase to nearly double (7.1 to 13.2% of cirrhosis hospitalizations) and nearly three quarters of those died within 90 days after hospitalization. These estimates of death include both hospital deaths and post discharge deaths. In our cohort, mortality for ACLF increased despite improvements of medical care. The significant increase in cirrhosis hospitalization over the study period is relate with various factors such as patient demographics, liver disease epidemiology, and clinical practice patterns. These factors can plausibly account for the drastic increase in cirrhosis hospitalization. Demographic changes, such as the aging population, with a high rate hepatitis C cirrhosis, have amplified the inferior survival in ACLF. The number of older patients with hepatitis C who live long enough to experience progression of their liver to hepatic decompensation have been increasing. Moreover, they were an evident trend related to obesity pandemic, leading to increasing prevalence of nonalcoholic fatty liver disease. Finally, the characteristics of infections in patients with cirrhosis have changed over the last decades, with a trend toward an increase in nosocomial infections, which are strong independent predictors of poor outcomes. This is important because two thirds of our ACLF cases had sepsis. This finding agrees with previous data showing that infections are a leading cause of hospitalization, decompensation, and death in patients with cirrhosis. Those data highlighted urgent needs for national policy and research for the care of patients with cirrhosis to prevent them from developing ACLF. The inpatient ACLF mortality was also associated with type and number of organ failures. Similarly, with the European and NIS studies [7, 11], we discovered that if patients developed more organ failures, then the mortality risk significantly increased. Cardiovascular, respiratory, and renal failures had the highest probability of mortality. Strategies to prevent ACLF-related mortality need to target high-risk patients. Furthermore, organ support and clinical care map of emergency liver transplantation needs development to standardize care of the sequence of ACLF patient care activities performed. Investigators of the Chronic Liver Failure Consortium have recommended early introduction of intensive care for ACLF patients within 7 days and ACLF grades assessment with different time points in order to define prognosis and assist decision-making for proper management continuation between liver transplantation and best supportive care [13].

Since care of hospitalized patients with cirrhosis, especially those with ACLF requires intensive care, so the cost of treatment of those are also expensive. However, there are only few worldwide studies of the economic burden of ACLF. Data from United States insurance payer databases showed that costs per patients-per-month of chronic liver disease associated with hepatitis C infection rose in relation to the severity of liver disease status with most involved with inpatient care. Among those with chronic liver disease associated with hepatitis C, end-stage liver disease patients have highest treatment costs [14]. Care of patients with ACLF trended to prolonged hospitalization because of intensive care unit requirements using multidisciplinary care teams with multiple-organ support. The cost of patients with cirrhosis and ACLF from NIS database from 2001 to 2011 showed that the mean cost per ACLF hospitalization in 2011 ($51,841) was around 4 times more compared with cirrhotic patients without ACLF ($14,894). The ACLF economic burden is possibly much larger if extra costs of rehabilitation facilities, nursing homes, and hospice are also taken into account. The use of Diagnosis-Related Group-Based (DRG) reimbursement for ACLF hospitals may not get adequately reimbursed. The disadvantage of DRG reimbursement is creating financial incentives toward earlier hospital discharges.

Our study had some limitations which are inherent to research involving administrative database analysis. First, this study relied on ICD 10-WHO codes to establish cirrhosis and ACLF diagnosis with short specific period (2009 to 2013). Miscoding or error in the code-assignment process could occur which may lead to misclassification bias. Second, our data from the National Health Security Office did not include full clinical details such as medications and laboratory data. Therefore, those included potential time-based patterns for diagnosing coding from ICD 10-WHO codes that would have affected our case definition. Nevertheless, this study has demonstrated the first nationwide prevalence and cost of ACLF hospitalizations outside of the United States. Our findings have demonstrated several important healthcare burdens of ACLF.

In conclusion, this study used a nationwide database to discover the burden of ACLF including both the natural course of the disease and also from a health economic perspective. Despite the inherent limitations of using administrative databases, this study’s results could have several clinical implications, especially for the potential to assist health care policy stakeholders to target high-risk patients. Future studies should focus on suitable strategies to target those patients with high benefit for liver transplantation or best supportive care and aim to reduce mortality in cirrhotic patients who develop ACLF.

## Supplementary Information

**Additional file 1: **Definition of cirrhosis and acute on chronic liver failure (ACLF) used ad inclusion criteria in the study and costs of cirrhosis and ACLF.

## Data Availability

The data that support the findings of this study are available on request from the corresponding author, [SC]. The data are not publicly available due to their containing information that could compromise the privacy of research participants.

## Abbreviations

ACLF: Acute on chronic liver failure
APASL: Asia Pacific Association for the Study of Liver
CANONIC: (CLIF Acute on Chronic Liver Failure in Cirrhosis)
CI: Confidence Interval
DRG: Diagnosis-related Group
HR: Hazard Ratio
ICD-10-CM: International Classification of Diseases, 10th Revision, Clinical Modification
NACSELD: North American Consortium for Study of End-Stage Liver Disease
NIS: National Inpatient Sample
WHO: World Health Organization
