# Supplementary material for: Health care burden and mortality of acute on chronic liver failure in Thailand: a nationwide population-based cohort study
Source: BMC Health Serv Res. 2022 Feb 7;22:156. doi: 10.1186/s12913-022-07574-6 (PMC8819862; doi:10.1186/s12913-022-07574-6)
Supplement: Supplementary file 1 — Additional file 1: Definition of cirrhosis and acute on chronic liver failure (ACLF) used ad inclusion criteria in the study and costs of cirrhosis and ACLF. [file 12913_2022_7574_MOESM1_ESM.docx]

**Supporting Table S1** Definition of cirrhosis and acute on chronic liver failure (ACLF) used ad inclusion criteria in the study

| **Cirrhosis** (any one of diagnoses below) | ICD-10 |  | **ACLF** (any CIRRHOSIS code plus of TWO organ failures below) | ICD-9 CM/ ICD-10 |
| --- | --- | --- | --- | --- |
| Alcoholic cirrhosis | K70.3 |  | **CARDIOVASCULAR** |  |
| Cirrhosis without alcohol | K74.0 |  | Central venous pressure | 89.62 |
| Portal hypertension | K76.6 |  | Pulmonary artery/wedge pressure | 89.64 |
| Hepatorenal syndrome | K76.7 |  | Arterial line | 89.61 |
| Esophageal varices | I85.0, I85.01, I85.1, I85.11 |  | Septic shock | R65.1, R65.3 |
| Hepatic encephalopathy | K72.9, K72.91 |  |  | R57, Y84.3, A48.3 |
| Spontaneous bacterial peritonitis | K65.0, K65.8, K65.9 |  | Sepsis | R65.0, R65.2, R659 |
|  |  |  |  | A400, A410, B95, B96, N390 |
|  |  |  | **RESPIRATORY** |  |
|  |  |  | Mechanical ventilation | 96.7, 96.70, 96.71, 96.72 |
|  |  |  | **RENAL** |  |
|  |  |  | Hemodialysis | 39.95 |
|  |  |  | Peritoneal dialysis | 54.98 |
|  |  |  | Acute kidney failure | N17.0, N17.1, N17.2, N17.8, N17.9 |
|  |  |  | **CEREBRAL** |  |
|  |  |  | Hepatic coma | B15.1, B16.2, B19.11, B18.1, B16.0, B18.0, B17.0, B17.2, B18.2, B17.8, B19.0, B19.21 |
|  |  |  |  |  |

**Supporting Table S2** Costs of cirrhosis and acute on chronic liver failure (ACLF)

Hospital costs and payment*

Hospital reimbursement by Thailand government’s diagnosis-related group (DRG) system*

*Costs demonstrate in Thai baht, US $1 = 32.7 baht (referenced November 2021)
